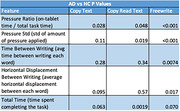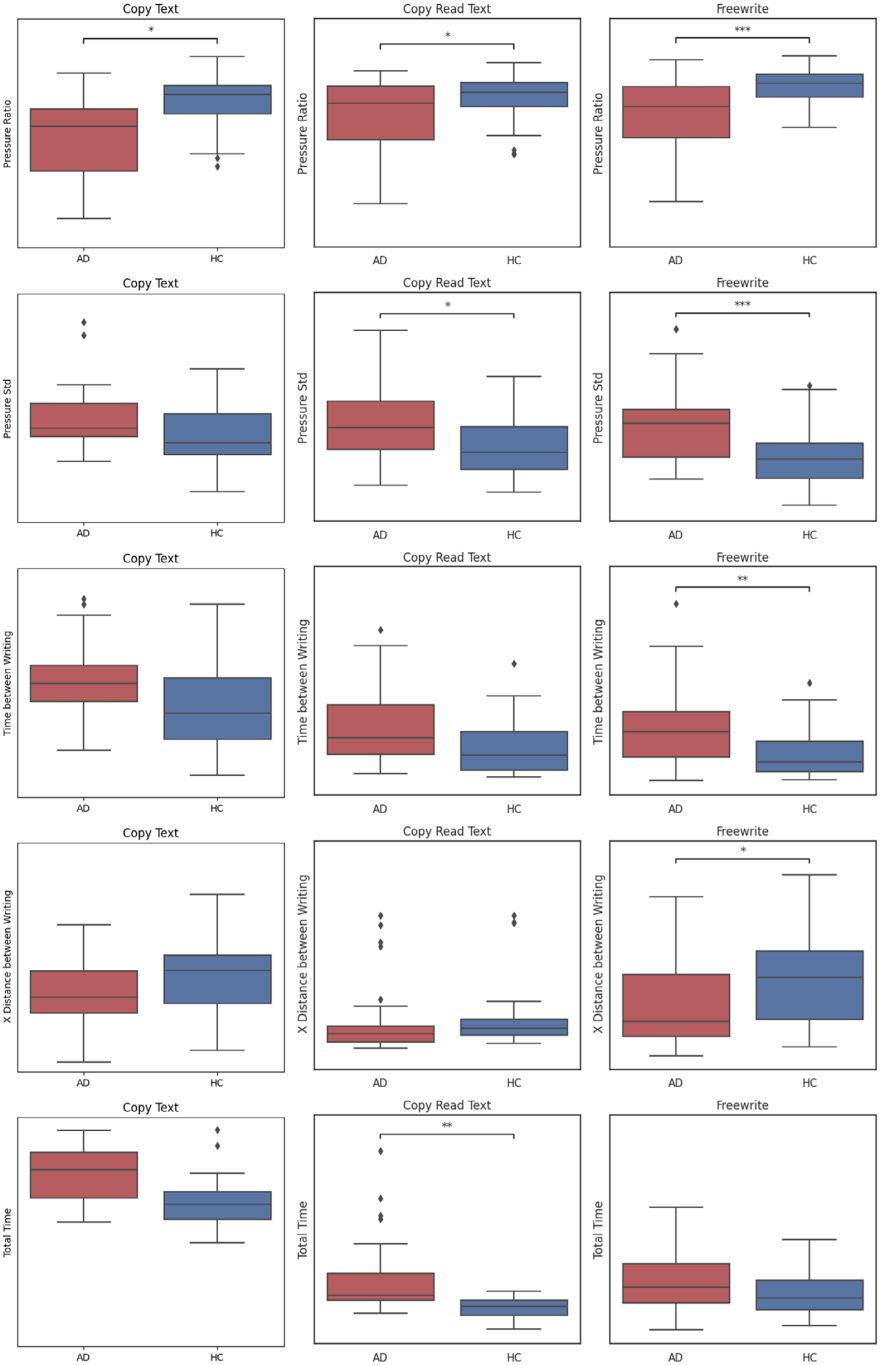# Cognitive Assessment through Writing Tasks

**DOI:** 10.1002/alz.093186

**Published:** 2025-01-09

**Authors:** Casey Chen, Thomas Thebaud, Ankur Butala, Najim Dehak, Laureano Moro‐Velazquez, Esther S Oh

**Affiliations:** ^1^ Johns Hopkins University, Baltimore, MD USA; ^2^ Johns Hopkins University School of Medicine, Baltimore, MD USA

## Abstract

**Background:**

Alzheimer’s Disease (AD) is a neurodegenerative disease which affects motor‐cognitive abilities including handwriting.

Past studies have used simple writing tasks to analyze handwriting fluidity, while more complex tasks can detect visual‐spatial deficits through spatial organization analysis. In this study, we extracted explainable features from the handwriting of AD patients performing various writing tasks using signal processing techniques and analyzed their importance in the cognitive assessment of AD.

**Method:**

We collected a dataset from 60 participants, 23 with AD or MCI due to AD and 37 controls (HC), using a digital tablet. The writing tasks used involved various degrees of freedom: In Copy Text and Copy Read Text participants copied a paragraph, with and without reading it out loud, adding a multi‐tasking complexity layer, and in Freewrite participants wrote a paragraph spontaneously. In‐air and on‐tablet movements and writing pressure were collected. Multiple explainable features were extracted, such as pressure standard deviation, writing time/completion time ratio, displacements between writing, and completion time. Features from AD and HC participants were compared using Welch’s t‐test and adjusted using the false discovery rate (FDR) correction.

**Result:**

The results show that the on‐tablet time/completion time ratio was significantly different between AD and HC (p‐value < 0.05) across all three tasks, and pressure variation was significantly different between groups for the Freewrite and Copy Read Text tasks. For Copy Read Text, there was a significant difference between groups in completion time. AD patients spent more time in‐air than on‐tablet with greater writing pressure variation.

For Freewrite, in‐air displacements between writing segments were significantly different (p‐value < 0.01) between groups. AD patients had longer average in‐air time and smaller horizontal displacement.

The results suggest a possible hesitation effect and a visual‐spatial deficit in AD patients.

**Conclusion:**

Analysis of writing tasks with varying complexity proved useful in assessing the motor cognitive abilities of AD patients. Our results show statistically significant differences between AD and HC participants for the on‐tablet time/total task time ratio and in‐air compared to on‐tablet times across all three writing tasks suggesting greater hesitance in writing among AD patients.